# Practical approach to last-mile converged free-space and fiber QKD for secure city-scale networks

**DOI:** 10.1038/s41598-025-34184-z

**Published:** 2026-01-28

**Authors:** Aristeidis Stathis, Argiris Ntanos, Panagiotis Kourelias, Evridiki Kyriazi, Panagiotis Toumasis, Nikolaos K. Lyras, Athanasios D. Panagopoulos, Hercules Avramopoulos, Giannis Giannoulis

**Affiliations:** 1 School of Electrical and Computer Engineering, National Technical University of Athens, 15780 Athens, Greece; 2 Optoelectronics Section, European Space Agency, Estec, Keplerlaan 1, 2201 AZ Noordwijk, The Netherlands

**Keywords:** Physics, Quantum physics, Quantum information

## Abstract

Current Free-Space Optical Quantum Key Distribution (FSO-QKD) systems are mainly focusing on the development of critical components needed towards the realization of satellite-based QKD, relying on bulky optical telescopes equipped with expensive optical systems. The cost and complexity of these systems hinder the establishment of a practical FSO-QKD infrastructure deployed as the wireless last-mile segment of Quantum Communication Infrastructure for applications in urban environments. In this work, we experimentally demonstrate successful transmission of polarization encoded qubits over 0.5 km free space metropolitan link with enhanced noise suppression for both nighttime and daylight operation. Βy deploying two low-cost, Single Mode Fiber-coupled, optical terminals along with a prototype quantum sender station operating in C-band, Quantum Bit Error Rate (QBER) values below 3.5% were achieved, with expected Secure Key Rates up to 1 kbps during nighttime. The FSO link was integrated with existing in-building fiber infrastructure, demonstrating stable operation and acceptable QBER values below 5.5%, even when coexisting with intense C-band light signals (> 10 mW) propagating alongside the photonic qubits. The field trial and its comprehensive verification paves the way for practical FSO-QKD in city-scale environments, enabling end users to share quantum keys over kilometer-scale wireless links between building rooftops in last-mile connectivity scenarios.

**Supplementary Information:**

The online version contains supplementary material available at 10.1038/s41598-025-34184-z.

## Introduction

**Fig. 1:**
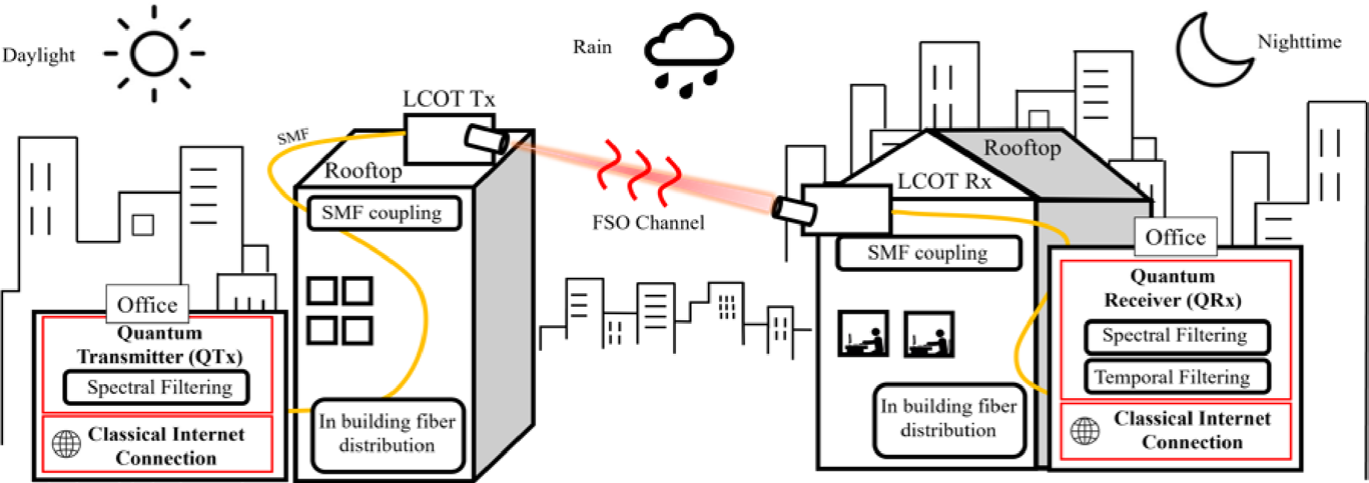
Envisioned terrestrial Fiber/FSO architecture in support of classical and quantum links.

Quantum Key Distribution (QKD) has seen rapid advancements, driven by growing interest from both the private and public sectors^1^. This surge in attention has accelerated the development of cutting-edge QKD prototypes^2–4^, with a strong focus on integrating these systems into existing telecommunication networks, paving the way toward the ambitious vision of a global quantum internet^5^. To achieve global connectivity quantum links must overcome inherent distance and scalability constraints. Significant efforts are underway to tackle long-range communication challenges, where photon loss and decoherence become critical, by advancing satellite-based systems^6^ and quantum repeater technologies^7^ to extend individual link distances. In contrast, fiber-based technologies efficiently manage scalability over shorter distances by leveraging the robustness and wide availability of existing telecommunication infrastructures^8^. Mature photonic components and optimized fiber networks enable high-rate, reliable key distribution in urban and regional settings, making them cost-effective and practical solutions where fiber connectivity is readily available. However, when faced with dense urban environments and limitations in physical space or high installation costs, fiber scalability is challenged, prompting the exploration of complementary solutions like free-space optical (FSO) links. Recent advances have overcome key obstacles in FSO technology, such as link alignment, atmospheric turbulence, mechanical instabilities, and daylight noise interference, making noise-sensitive quantum communication protocols like QKD^9^, MDI-QKD^10^, and entanglement distribution^11^ ready for deployment. While various demonstrations have showcased the potential of these systems to meet the growing demand for secure metropolitan communications, many rely on expensive, state-of-the-art equipment, such as Superconducting Nanowire Single-Photon Detectors (SNSPDs)^12^, specialized daylight noise filters^13^, and high-end optical terminals^14^ tailored to specific infrastructures. Furthermore, a significant number of FS-QKD experiments have focused on advancing technology for satellite deployment^9,10,15–17^, leaving the challenges of readily deployable terrestrial networks unaddressed. This gap could possibly stem from FSO technology lagging behind fiber-based systems in technological maturity, particularly in the availability of off-the-shelf, plug-and-play solutions essential for real-world deployment. In this work, we present a practical FS-QKD system that fills this gap by leveraging relaxed system requirements and widely available and affordable Commercial Off-The-Shelf (COTS) components. Our implementation features a 20 MHz repetition rate polarization-encoded QKD source operating within the C-band, a wavelength choice that benefits from lower solar spectral irradiance and reduced Rayleigh scattering compared to visible and near-infrared light but also enables coupling into Single Mode Fiber (SMF), facilitating seamless fiber-FSO-fiber convergence in order to leverage the existing telecom infrastructure. The system is built on Low-Cost Optical Terminals (LCOT) with integrated fiber coupling, eliminating the need for separate fiber coupling modules, along with SPAD detection. Exploiting a novel combination of spatial and temporal filtering techniques, the presented FSO-QKD system can support the classical/quantum coexistence transmission over fiber segments used for distribution in-building the quantum signals as shown in Fig. 1. Despite these relaxed requirements, our implementation successfully enabled both nighttime and daytime QKD. Specifically, field trials over a 0.5 km FSO link in Athens demonstrated QBER values below 3.5% and Secure Key Rates (SKR) of up to 1 kbps. Additionally, fiber coupling enabled us to position the Quantum Receiver (QRx) away from the LCOT (200 m from the rooftop to the lab in our case)—demonstrating the potential to extend communication distances and securely route key material to enclosed spaces such as offices, where cryptographic keys are practically needed. This approach supports city-scale quantum networking without requiring trusted nodes to be on the city’s rooftops. Our implementation also demonstrated the ability to share infrastructure for both classical communication and QKD by enabling the coexistence of the C-band quantum wavelength with intense classical signals, such as clock synchronization and classical data traffic streams. To assess its robustness, we tested the system by introducing high-intensity classical light over the FSO channel, achieving acceptable QBER values and an estimated SKR of 500 bps even with + 10.7 dBm of launched classical optical power. These features enhance the feasibility of integrating QKD into real-world telecom networks while addressing key scalability and deployment challenges in an affordable and easy manner. Our approach enables practical and cost-effective hybrid free-space/fiber QKD networks that can be seamlessly deployed over metropolitan rooftops to deliver symmetric quantum keys. By addressing key scalability and deployment challenges, it enhances the feasibility of integrating QKD into real-world telecom networks in an efficient and affordable manner.

## Results and discussion

### Field-trial results

To evaluate the performance of our FSO QKD system, we conducted a two-day experimental campaign at the National Technical University of Athens (37.97° N, 23.78° E.), testing both nighttime and daytime operation during the 4th and 5th of July 2024. Hourly, the average coupling efficiency from transmitter (Tx) to receiver (Rx) collimator varied from 6% down to 0.5% where manual tip/tilt correction was applied to restore the FSO link’s efficiency to the maximum value. During the experiments, the clock (CLK) signal coexisted with the quantum pulses over the 0.5 km FSO link. The absence of active stabilization/steering combined with variable weather conditions (e.g., wind speed) impacted the setup’s receiver’s efficiency, leading to non-continuous data collection throughout the 48 h experimental campaign. In what follows, the experimental results on the performance of classical and quantum signals during transmission over the FSO channel are presented in detail, based on data collected from both night and day experimental campaigns.

## Nighttime operation

**Fig. 2:**
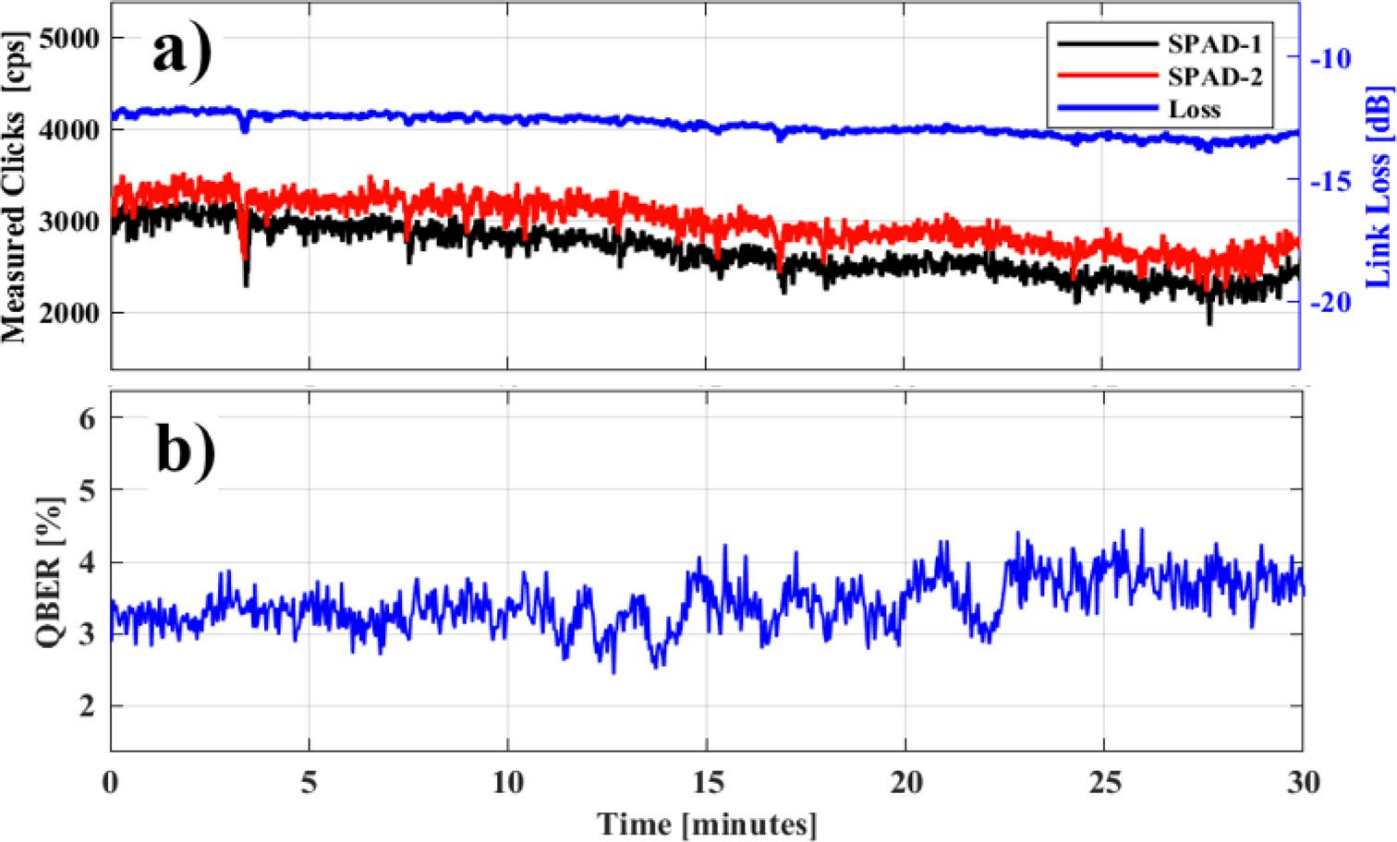
**a** Link loss (blue line) and measured click rate for each SPAD (red and black line), **b** QBER over time during the night.

For the quantum transmission quality assessment, the raw click rates were measured to calculate the QBER, as described in the Methods sub-section *Quantum Receiver (QRx)*. Figure 2. a presents the individual raw click rates of each Single Photon Avalanche Detector (SPAD) over the 0.5 km FSO link. The total raw click rate was measured at approximately 6 kcps, with nearly 3 kcps corresponding to the quantum signal detected by each SPAD. Additionally, 112 cps were attributed to noise, including contributions from the CLK signal (40 cps due to x-talk and 2 cps due to Raman noise) and the Dark Count Rate (DCR) of the detectors (approximately 35 cps for each SPAD in gated mode). The blue line in Fig. 2a represents the channel loss, which was monitored throughout the experiment and found to fluctuate between 12 and 13 dB. On the receiver side (QRx), the FSO channel was monitored by tapping a portion of the CLK signal using a 95:5 fiber splitter. This allowed us to calculate the channel’s scintillation index and characterize its turbulence strength. During nighttime, the scintillation index was determined to be σ_I_^2^ = 4.3 × 10^-2^, indicating a weak turbulence regime resulting in negligible scintillation losses. In parallel, we conducted a theoretical loss analysis, to estimate the contribution of the remaining loss components as detailed in the Methods section. Specifically, geometric attenuation accounted for approximately 2.1 dB, while SMF coupling losses added around 6.3 dB. Additionally, since Quantum Transmitter (QTx) was placed behind enhanced double-glazing window an extra 3 dB loss was introduced. These sum to 11.4 dB, which is 0.6–1.6 dB short of the total 12–13 dB loss measured experimentally. This could be attributed to additional minor losses unaccounted for, such as atmospheric absorption (~ 0.1 dB), intrinsic loss of LCOT (lenses, coatings), or unmodeled system inefficiencies such as instantaneous misalignment errors. Figure 2. b illustrates the average QBER values on the X basis obtained during nighttime, which ranged from 3.2 to 4%. To assess the performance of the QKD system over the FSO channel, the SKR serves as a key figure of merit. In this work, we estimate the system’s SKR under the specified conditions using an asymptotic approximation, as outlined in^18^, which accounts for the implementation of a decoy-state BB84 protocol. Further details of our methodology are provided in the Methods section. By incorporating experimentally measured parameters — specifically noise clicks ($\:{Y}_{0}$) and end-to-end link efficiency ($\:{\eta\:}_{channel}$) — into our theoretical model, we achieved a good agreement between the model’s predictions and the experimental results, with raw click rates agreeing within 3% and QBER values within 1%. Consequently, we estimated the corresponding SKR values, which were found to be 1032 bps, based on the experimentally measured raw click rates and QBER values. It is important to note that we chose to segment the data into 30-minute uninterrupted chunks since manual tip/tilt correction had to be performed regularly.

## Classical/Quantum coexistence

To evaluate the performance of our deployment under classical/QKD coexistence, we multiplexed along with the quantum (1550.12 m) and CLK signals (1548 nm) a CW laser also emitting at C-band centered at 1552 nm. The coexistence evaluation experimental campaign was also performed under nighttime conditions. Table 1 summarizes the impact of the classical laser on SKR and QBER, while also providing a breakdown of noise contributions. In addition to the theoretically estimated QBER, an experimentally measured QBER column is included, demonstrating strong agreement within ± 0.3% accuracy. Although crosstalk (X-talk) noise was significantly suppressed through a triple-stage 100 GHz band-stop filter (center at the quantum passband) applied to both the CLK and CW signals, along with two bandpass filters at the receiver side, it remained the primary contributor to the rising QBER. Note that the CW intensity value provided in Table 1 refers to the output of the laser source on the QTx side. The optical power entering the fiber is lower, as it is attenuated by the loss of the FSO link. Raman noise from the 200 m fiber was measured as detailed in Supporting Information. Overall noise click rates of 112 cps (CW Intensity off) and 450 cps (CW Intensity of up to + 10.7 dBm) were attributed to Raman noise (2–80 cps) and crosstalk noise (40–300 cps), respectively.

**Table 1:** SKR, QBER and noise parameters values.

CW Intensity (dBm)	SKR (bps)	QBER (%)		Noise (cps)		
		Theoretical	Measured	DCR	X-talk	Raman
Off	1013	3.2	3.5	70	40	2
+ 5.7	794	3.74	3.7	70	110	20
+ 7.7	645	4.1	3.9	70	150	30
+ 9.7	501	4.47	4.5	70	180	50
+ 10.7	90	5.35	5.1	70	300	80

**Fig. 3:**
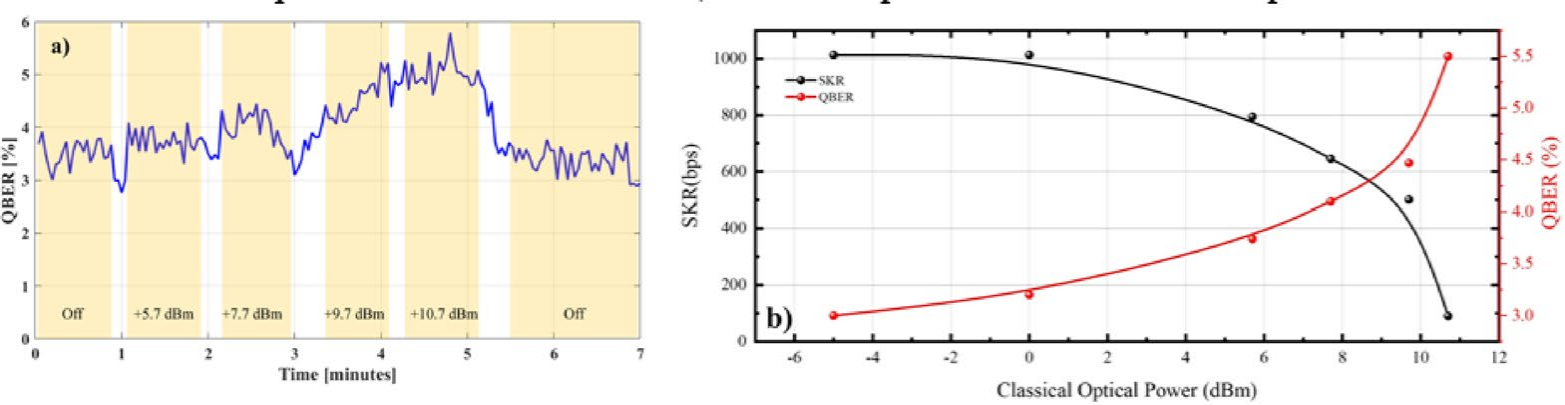
**a** Experimental QBER and **b** Theoretical QBER & SKR for various launched optical powers over the FSO channel.

Figure 3. a shows the experimentally measured QBER values as the power of the classical laser is increased over time. During each change in classical intensity, a brief transition period is required for the laser to stabilize at the new intensity level. This process takes approximately 3 s, and as a result, data recorded during these transitions may exhibit temporary drops in the QBER. The regions that have been used for the calculation of the mean QBER values for each intensity level, are highlighted in Fig. 3a. The FS-QKD link maintained QBER values below 6%, even with + 10.7 dBm of launched optical power, whereas for launched intensities of 0 dBm or lower the QBER value remained practically unaffected by the classical intensity. The corresponding estimated SKRs shown in Fig. 3b ranged from 90 bps at + 10.7 dBm to approximately 1.0 kbps when 0 dBm (or less) of classical power was transmitted through the FSO link. This highlights the resilience of the FSO channel compared to fiber transmission, as similar power levels in a fiber-optic channel with comparable length would typically generate Raman photons that strongly contaminate the quantum signal, significantly degrading system performance. Unlike fiber, where Raman scattering is an inherent and unavoidable nonlinear effect, the FSO channel mitigates this issue due to its free-space propagation, which lacks the same nonlinear interactions. This makes FSO a promising alternative for quantum communication, especially in scenarios where high-intensity classical signals must coexist with quantum signals.

## Daytime operation

In what follows the results from the field trial during the day are presented. Daytime FSO QKD presents an additional challenge due to the increased noise caused by sunlight. The bright background light significantly raises the photon noise level, making it difficult to distinguish the weak quantum signals from the surrounding noise. In this work, we employed temporal and spectral filtering techniques along with SMF coupling to reduce the excess noise by sunlight in order to be able to perform valid measurements. More specifically, to isolate the quantum passband, dedicated at 1550.12 nm, a two-stage Band-Pass Filter (BPF), consisting of a 12.5 GHz spectral slice of a Flex grid Wavelength Selective Switch (WSS), with IL = 4.5 dB (Finisar DWP-EK-AA, Suppression Ratio (SR) = 40 dB) cascaded with a 25 GHz DWDM BPF (OPNETI C34 ITU-grid 25 GHz DWDM, IL = 1.5 dB, SR = 30 dB), were used. Despite its higher loss, the WSS was preferred instead of a second BPF due to its high SR and stable operation at a very narrow passband. In addition, we complemented our spectral filtering with temporal filtering, by operating the SPADs in Geiger mode with a time window equal to 6 ns. Finally, SMF coupling reduced background solar radiance noise by acting as a spatial filter, allowing only light that matches the mode field diameter of the SMF to pass through. Therefore, SMF-coupling strongly rejects stray light which often spread across multiple spatial modes by a factor of several hundred as discussed in detail in the Methods section. In addition, the narrow acceptance angle of the fiber further limits unwanted light from entering the system, improving the signal-to-noise ratio. However, it was observed that the primary contribution of solar background noise was coupled directly into the SMF segment exposed to sunlight, rather than through the collimator. In a practical deployment scenario, this issue could be effectively mitigated by using optical fibers with special coating to prevent ambient light from coupling into the fiber core. Since such specialized fibers were not available for our experiment, we implemented a combination of spectral and temporal filtering techniques to suppress background noise during the day. Specifically, the spectral filtering discussed above resulted in a reduction in the background noise from 17 kcps down to just 1100 cps when SPADs operated in free running mode. Temporal filtering was also applied to mitigate background noise by restricting the detection window to 6 ns. Before filtering, the detected signal was 1100 clicks per second, and after applying the temporal filter, this value decreased to 230 clicks per second. A detailed breakdown of the noise contributions is shown in Table 2 below:

**Fig. 4:**
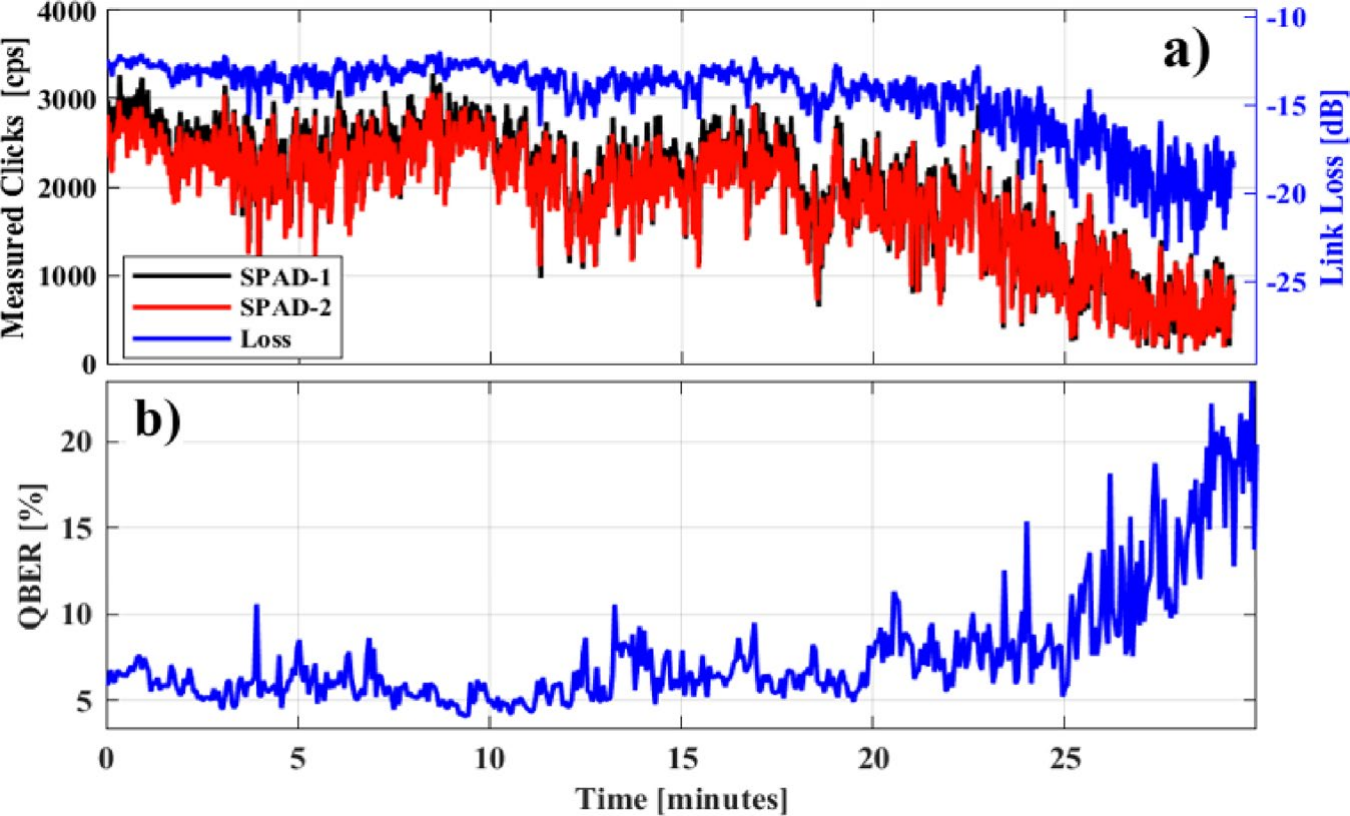
**a** Link loss (blue line) and measured click rate for each SPAD (red and black line) **b** QBER over time during the day.

**Table 2:** Breakdown of noise contributions for daylight operation.

Measurement Condition	Noise Click Rate	Breakdown
Unfiltered	17,000 cps	Neither spectral nor temporal filtering applied.
Spectrally filtered	1100 cps	Only spectral filtering applied (2 BSFs at QRx).
Total noise (Spectrally and temporally filtered)	230 cps	SPADs DCR = 2 × 35 cps (@ gated mode), CLK noise: 40 cps due to X-talk, 2 cps due to SpRS Sky radiance coupling directly to fiber from collimator ~ 10 cps Exposed fiber collecting ambient light ~ 108 cps

After dealing with the background noise, we proceeded to establish the daylight FS-QKD link. In general, the conditions to establish the 0.5 km FSO link during the day were much more challenging compared to the nighttime operation due to winds during that day. In contrast to the nighttime, daytime measurements showed a higher scintillation index of σ_I_^2^ = 5 × 10^− 2^, also reflecting weak turbulence regime. These measurements also align with the higher wind speed on the 5th of July as calculated from the ECMWF ERA5 Reanalysis (Copernicus Climate Change Service, 2017) retrieved from^20^. More details regarding the wind speed over the two-day experimental campaign are provided in Fig. S4 in Supporting Information Online. After reaching stable coupling efficiency and achieving losses comparable to those of the nighttime operation, we recorded the raw click rates and the QBER values during daylight continuously for 30 min as depicted in Fig. 4a. The link loss exhibited stronger fluctuations while its average value remained relatively stable for the first 20 min. The channel faded much quicker than in the nighttime, which can be seen in Fig. 4a after 20 min. The channel fading is mainly attributed to QTx-QRx misalignment due to the stronger wind speed (almost doubled compared to nighttime operation), since one of the two optical terminals of the FSO link was installed in outdoor environment. Despite that, QBER values were within an acceptable range over the first 20 min and strongly increased as the channel began to fade shown on Fig. 4b.

**Fig. 5:**
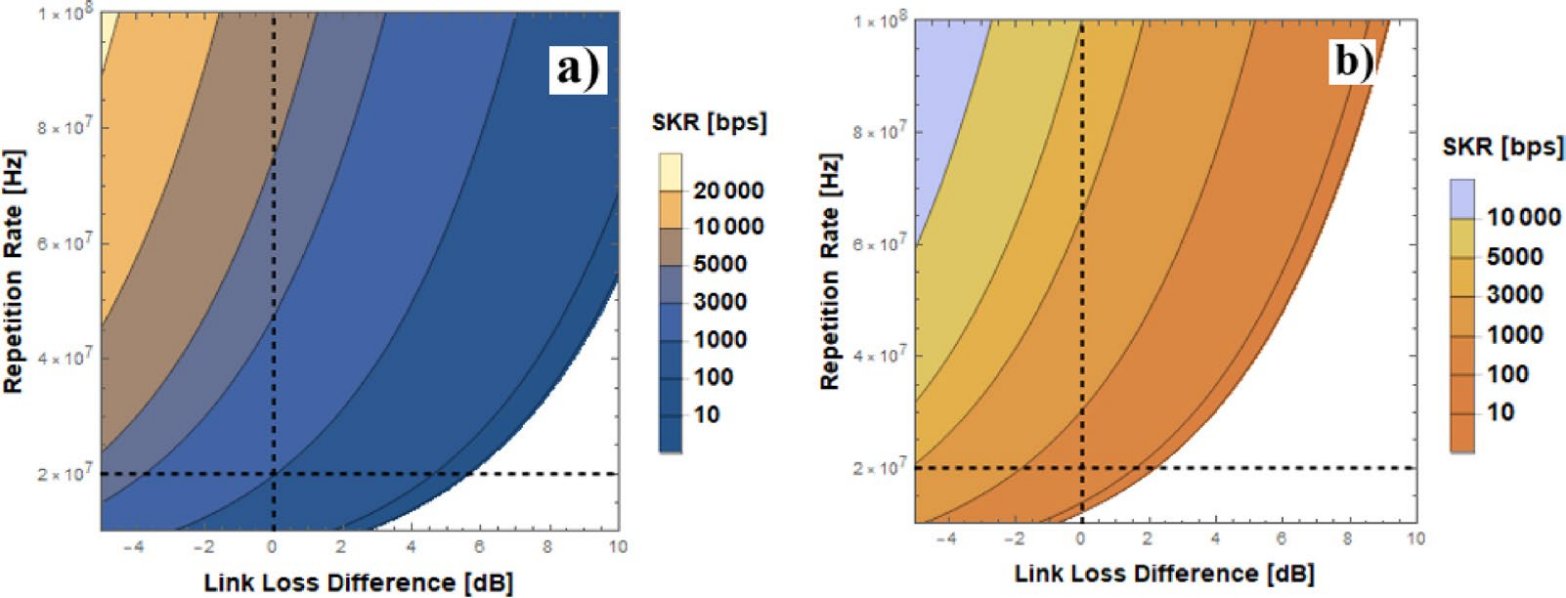
Contour plot of calculated SKR values over different source’s repetition rates and link loss for **a** Nighttime conditions and **b** Daylight conditions.

By following the same methodology as discussed in the nighttime section and accounting for the measured raw click rates of both SPADs, SKRs of up to 350 bps were estimated. Figure 5a, b presents a contour plot of the expected SKR over the FSO link as a function of the source’s emission rate and the link loss difference for nighttime and daytime conditions, respectively. At 0 dB, the link loss corresponds to the value measured in our experimental testbed, while lower values indicate reduced loss (e.g., if larger optical terminals are used), and higher values represent increased loss (e.g., due to greater link distances or harsher atmospheric conditions). The intersection of the vertical and horizontal dashed lines marks the operating point of our experiment. At this point the experimentally measured value of the QBER in the signal state and the signal gain match the theoretically determined values. Notably, for the source repetition rate used in our setup (20 MHz), the system can tolerate approximately 5 dB of additional loss in nighttime conditions and 2 dB in daylight before the SKR drops to zero. It should be noted that the detection time window was set to 12% of the pulse period, consistent with our experimental setup (6 ns at 20 MHz), to ensure equivalent temporal filtering across different repetition rates. As a result, the noise level in counts per second was considered independent of the emission rate, assuming the detection window scales accordingly. However, if a higher emission rate (e.g., 100 MHz) is employed under the same link loss conditions, the system not only achieves a SKR of nearly 10 kbps but also exhibits significantly greater loss tolerance, enhancing robustness against atmospheric effects.

## Methods

This section outlines the experimental setup deployed and the methodologies followed during the field trial. A detailed description of the QKD sub-components, FSO terminals, and the specific instruments used is provided to ensure the reproducibility of the study. Our modelling regarding the FSO channel as well as the QKD protocol is also presented.

### Quantum transmitter (QTx)

**Fig. 6:**
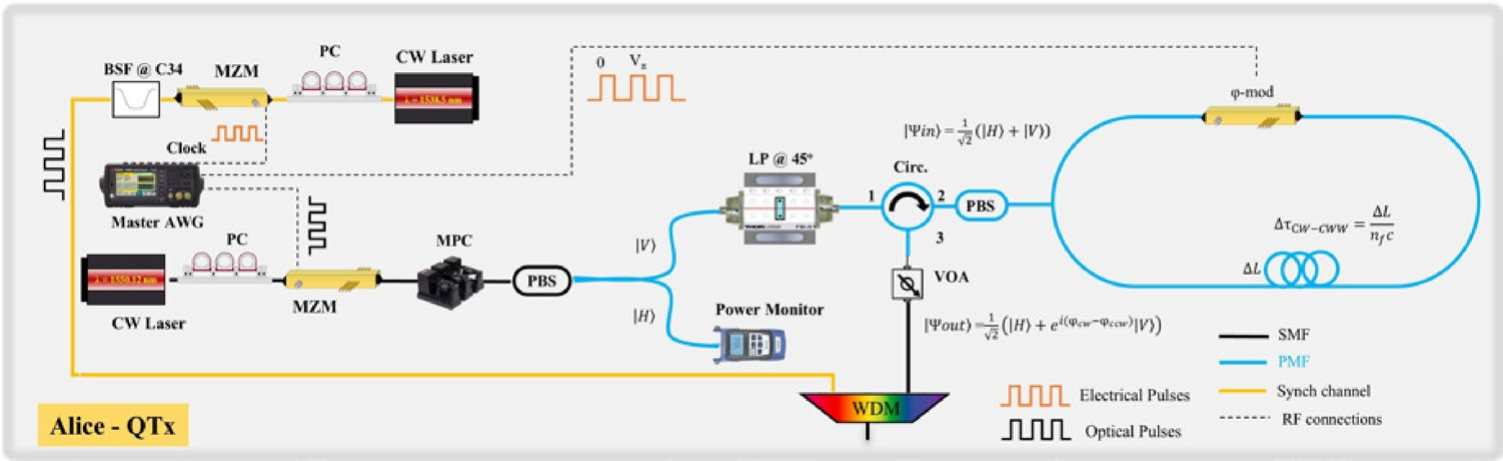
Schematic representation of the 20 MHz Polarization-encoding Sagnac-based Quantum Transmitter (QTx) setup.

The QTx employed, depicted in Fig. 6, is an in-house built prototype employing the standard polarization-encoded BB84 protocol and is based on a Sagnac interferometric configuration like^21–23^. In detail, the QTx consists of a Continuous Wavelength (CW) C-band narrow linewidth laser (< 100 kHz linewidth) emitting at 1550.12 nm. The CW laser is carved into 5 ns pulses at a repetition rate of 20 MHz via a Mach-Zehnder Modulator (MZM) driven by an Arbitrary Waveform Generator (AWG Keysight 33600 A). The QTx emission rate was practically limited by the driving electronics (with a minimum pulse duration of 5 ns), although the photonic blocks (the pulse carver and the Sagnac-type state encoder) can support significantly higher rates. The laser emits vertically polarized light which is further filtered regarding its polarization by passing through a Polarization Beam Splitter (PBS). A Motorized Polarization Controller (MPC) ensures that the polarization extinction ratio remains above 30 dB, while the power is monitored in the second port of the PBS to maintain a stable operation regarding the mean photon number per pulse. The vertically polarized pulses are then launched into free space through a free space polarizer rotated at a 45 degree to create an equal superposition of $\left| H \right. >$ and $\left| V \right. >$ polarization states. The free space polarizer is chosen instead of fiber due to the intrinsic stability of polarization in free space. After the polarization preparation, the light is coupled back into a single-mode optical fiber for further transmission through the system. In order to modulate the polarization, the pulses are injected through a Polarization Maintaining (PM) circulator into a PM PBS which marks the start of a Sagnac loop. The light is split equally into the clockwise and counterclockwise modes of the loop. The Sagnac loop incorporates a LiNbO_3_ Phase Modulator (PhM) in which the pulses are inserted with a polarization state that is diagonal with respect to the optical axis of the modulator’s crystal. By carefully timing the clockwise and counterclockwise pulses and by applying specific voltage levels, the ordinary and extraordinary refractive indices of the crystal vary independently, allowing to control the relative phase between the H and V polarization. Detailed information about the interferometric scheme described above can be found in^21^. Thus, at the output of the circulator it is possible to prepare two mutually unbiased polarization basis just by tuning the phase of the PhM, and that includes also the four polarization states of the standard BB84 protocol i.e.,

1 $$\left. {\left| D \right.} \right\rangle = \frac{1}{{\sqrt 2 }}\left( {\left. {\left| H \right.} \right\rangle + \left. {\left| V \right.} \right\rangle } \right),\left. {\left| A \right.} \right\rangle = \frac{1}{{\sqrt 2 }}\left( {\left. {\left| H \right.} \right\rangle - \left. {\left| V \right.} \right\rangle } \right)$$

$$\left. {\left| L \right.} \right\rangle = \frac{1}{{\sqrt 2 }}\left( {\left. {\left| H \right.} \right\rangle + i\left. {\left| V \right.} \right\rangle } \right),\left. {\left| R \right.} \right\rangle = \frac{1}{{\sqrt 2 }}\left( {\left. {\left| H \right.} \right\rangle - i\left. {\left| V \right.} \right\rangle } \right)$$

In our implementation, polarization modulation was performed using predetermined patterns, specifically sequences of alternating |D〉 and |A〉 or |R〉 and |L〉 polarization states. This was accomplished by driving the PhM between two distinct voltage levels, while the modulation frequency was synchronized with the emission rate of the MZM. The intrinsic QBER (iQBER) of our source was measured to be lower than 1%. More information can be found in Supplementary Information Online whereas iQBER measurements are shown in Fig S2. After port 3 of the circulator, where the polarization modulated signal is received, the optical power level is carefully adjusted to achieve the desired mean photon number µ = 0.65 through cascaded attenuation stages. The intensity of the modulated optical signal is measured with an optical power meter to −37.7 dBm before adding another 50 dB with a fixed attenuation stage. Therefore, the overall optical intensity is considered to be ~ −87.7 dBm, which corresponds to a mean photon number of about 0.658 photons per pulse. Both classical (clock) and quantum signal are combined and transmitted through the same medium using a C34 and a C36 ITU-grid Dense Wavelength Division Multiplexing (DWDM) 100 GHz modules, with a total Insertion Loss (IL) of 0.5 dB and a Suppression Ratio (SR) of 25 dB.

## Quantum receiver (QRx)

**Fig. 7:**
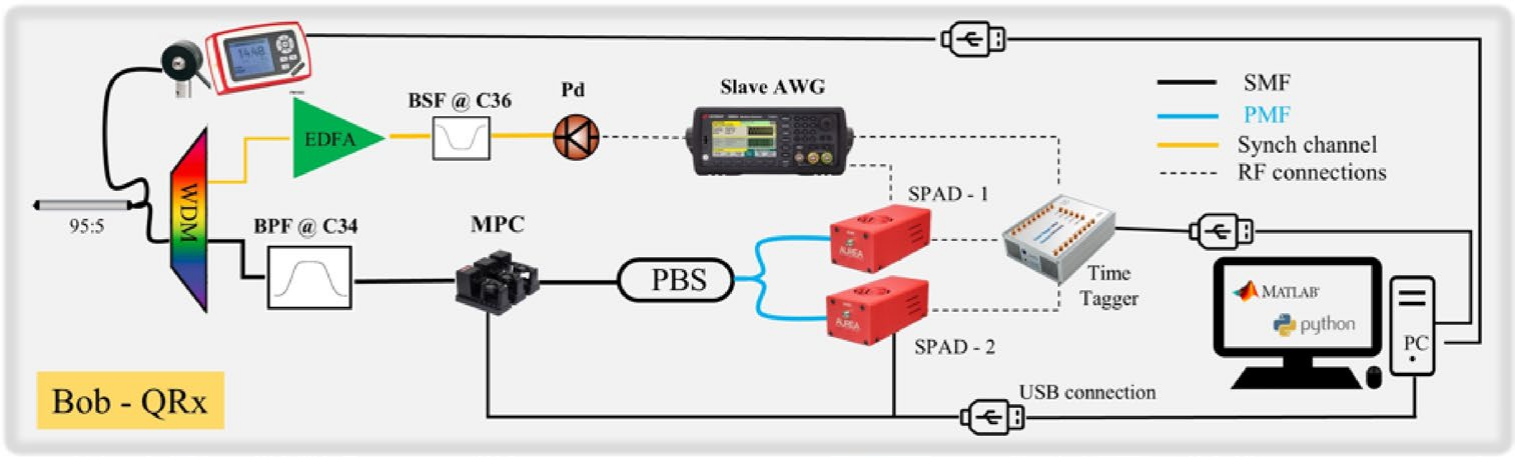
Schematic representation of the QRx optical setup including the Polarization analysis and the clock recovery modules.

The QRx is shown in the Fig. 7 below. Since the classical clock and the transmission of qubits are conveyed through the same medium, i.e., either fiber or free space, the same 100 GHz DWDM modules were used to demultiplex the signals. In addition, to further isolate the quantum passband a two-stage band-pass Filter (BPF), featuring cascaded passbands of 0.1 nm (Finisar DWP-EK-AA, IL = 4.5 dB, SR = 40 dB) and 0.2 nm (OPNETI C34 ITU-grid 25 GHz DWDM, IL = 1.5 dB, SR = 30 dB) respectively with a total loss of 6 dB, was used on the receiver side. Regarding the polarization analysis module, the incoming photons are measured by performing projective measurements in either the X (D/A polarizations) or the Υ (R/L polarizations) basis. The projective measurements are implemented using an MPC and a PBS for each basis. The MPC is also used to mitigate any polarization drifts due to the non-PM fiber patch cords in the setup. Since the polarization modulation was predetermined, the projective measurements were performed by rotating the MPC to the corresponding position, ensuring projection onto the correct basis each time. The estimation of the QBER in each basis is performed using a time-tagger (Swabian Instruments GmbH) in which the time-tags correspond to the photon’s arrivals. The time tagger receives a reference Transistor-Transistor Logic (TTL) pulse at 20 MHz generated by the slave AWG to keep track of the corresponding timestamps of each bit. Each timestamp corresponds to one of the four polarization states depending on the predetermined pattern. Finally, the data are processed using a custom software code that calculates the QBER_X_ and the QBER_Y_ using the following equation:

2 $$\:QBER\:=\frac{Fals{e}_{clicks}}{Correc{t}_{clicks}+Fals{e}_{clicks}}.$$

The SPAD units (AUREA OEM_NIR) used, operated with $\:qe=10\%$ quantum efficiency in gated mode (6 ns gate window), with a total dark count of about 35 cps (gated mode) and at a 20 µs dead time.

## Time synchronization

**Fig. 8:**
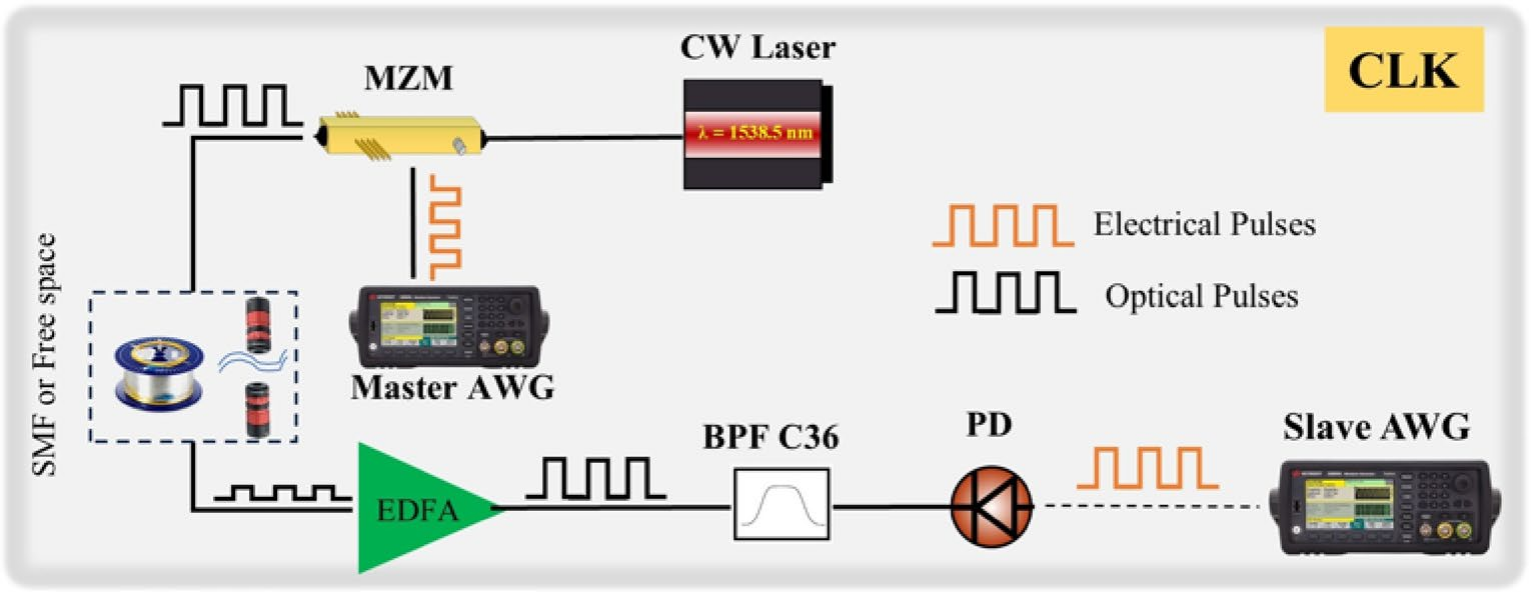
Schematic representation of the QTx and QRx synchronization setup.

The time synchronization is provided by a typical optical clock recovery scheme, depicted in Fig. 8, which utilizes two AWGs. All the electronics signals that were used to drive the MZI, the PM inside the Sagnac loop, as well as the SPADs and the Time Tagger were generated by these two AWGs. In detail, the 10 MHz TTL internal reference clock signal provided by the “master” AWG was used as a driving signal of an intensity MZM, to carve the CW laser emitting at 1548 nm (C36-ITU) used for the clock signal. In the QRx side, after the demultiplexing, the clock signal was amplified using an Erbium Doped Fiber Amplifier (EDFA) and was retrieved via direct detection using a 10 GHz Linear InGaAs PIN + TIA Optical Receiver. To filter the broadband noise from the EDFA, a 100 GHz C36 ITU-grid BPF was used. By feeding the retrieved signal into the external input of the “slave” AWG the clock can be recovered, thus enabling the timing locking between the two AWGs.

### The field trial and FSO channel characterization

**Fig. 9:**
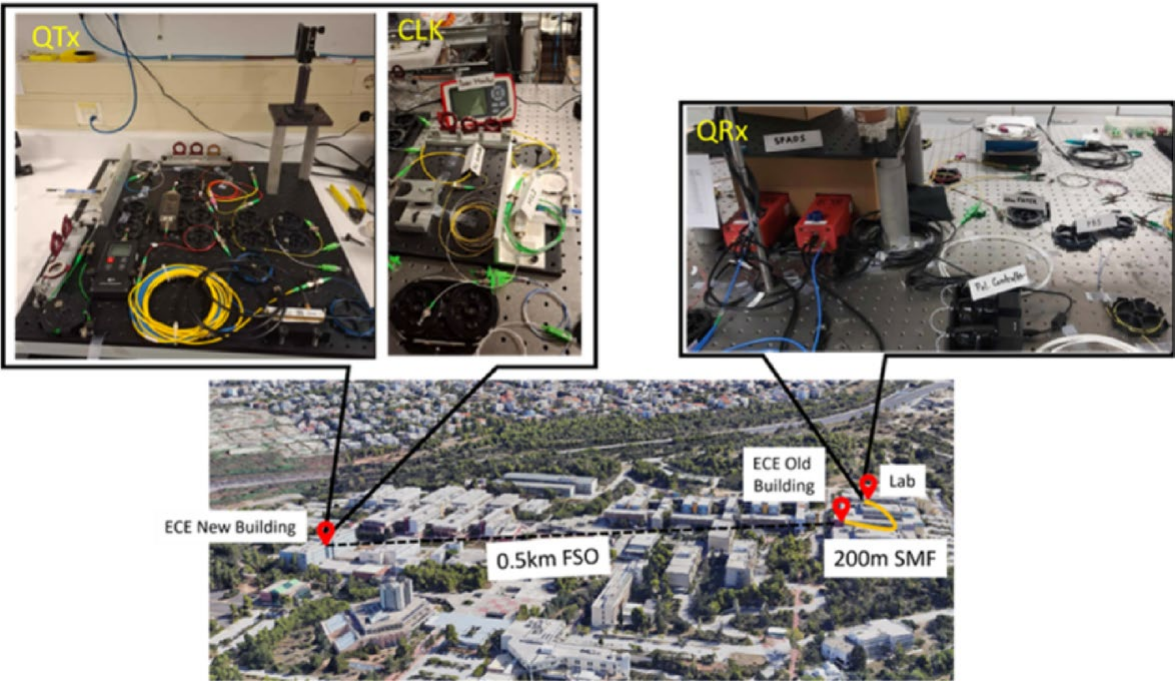
Free space optical link in the campus of National Technical University of Athens, interconnecting old and new buildings of the School of Electrical and Computer Engineering. *Source*: Map data © Google, *Google Maps* [2025]. Retrieved February 2025, from https://www.google.com/maps

A two-day comprehensive FSO QKD demonstration was conducted, spanning from July 4th to July 5th, 2024, encompassing both daytime and nighttime operation. The QTx (Alice) and QRx (Bob) were placed at the New and Old Buildings of the School of Electrical and Computer Engineering respectively, inside the campus of the National Technical University of Athens shown in Fig. 9. Alice and Bob were connected through a half kilometer FSO link. To establish the 0.5 km FSO link, each optical terminal employed a lightweight (0.5 kg), compact 42.5 mm air-spaced achromatic fiber collimator (Thorlabs C80APC-C, ARC 1050–1650 nm), whose fiber-coupled design provides a small physical footprint (≈ 13 × 5.6 × 5.6 cm³) and relatively low cost (~€1.4 k), in strong contrast to the large telescope assemblies or custom optomechanical structures commonly used in free-space QKD terminals. Both collimators were mounted on two-adjuster kinematic mounts with an angular adjustment range of ± 3°. Each collimator has an 80 mm effective focal length to the fiber while the beam can be focused between the maximum waist distance and the closest focusing distance thus allowing for optimization of coupling efficiency from free space into a fiber. For the alignment a beacon laser at 650 nm was used. The total attenuation introduced by the FSO channel was measured to be around 12.5 dB (mean value). The absence of active stabilization/steering along with weather conditions impacted the FSO link efficiency, leading to non-continuous data collection throughout the 48 h experimental campaign. The average FSO link losses from collimator to collimator fluctuated between 12 and 26 dB on an hourly basis. Manual tip/tilt correction was applied as needed to restore the link’s efficiency to its typical operating level. The full-scale setup is depicted in Fig. S1 in Supplementary Information Online. At the sending terminal the QKD photons were prepared as described above and were sent together through free space, coexisting with the clock signal, through the 42.5 mm collimator. These photons are coupled into a SMF for spatial filtering from the Rx collimator and transferred through one in-building single 200 m SMF in the lab, with an additional 1 dB loss (due to the multiple connection points). Both classical and quantum photons are transmitted through a DWDM, matched with the one on the QTx for spectral filtering while the QKD photons are sent into the BB84 analysis module, and the clock signal is sent to the clock recovery scheme as depicted in Fig. 8. To characterize the turbulence strength over the half kilometer FSO channel the received clock’s optical power was monitored via a 95:5 monitor port. A parameter for the strength of intensity fluctuations is the normalized variance of the intensity—usually called the intensity scintillation index ($\:{\sigma\:}_{I}^{2}$ ) and is given in Eq. (3)^24^:

3 $$\:{\sigma\:}_{I}^{2}=\frac{{<I}^{2}>}{{<I>}^{2}}-1,$$

where *I* is the received optical irradiance and < > denotes ensemble average. The value of the $\:{\sigma\:}_{I}^{2}$ can be directly calculated from the recording of the intensity over time, via the 95:5 beam splitter of the clock signal. The clock intensity recording has been performed with a sampling rate of 100 Hz. More information about the methodology followed can be found in Fig.S3 in Supplementary Information Online.

### Channel modeling

In what follows, a detailed description of the channel modelling is provided. The modelling considers the effect of atmospheric absorption, receiver collection efficiency as a function of beam broadening, beam wandering and atmospheric scintillation, and SMF-coupling in the presence of atmospheric turbulence to calculate the overall FSO channel loss. In this work, some of these parameters have been experimentally measured and they are then compared to those of our theoretical predictions. The total channel efficiency is measured based on the clock intensity monitoring via the 95:5 beam splitter, while optics efficiency, filter efficiency, fiber optics efficiency, and detector efficiency are also experimentally measured. The overall free-space channel efficiency is finally calculated by the following equation:

4 $$\:{\eta\:}_{channel}=\:{\eta\:}_{atm}{\eta\:}_{{R}_{x}}{\eta\:}_{SMF}{\eta\:}_{turb},$$

where $\:{\eta\:}_{atm}$ denotes the atmospheric channel absorption, $\:{\eta\:}_{{R}_{x}}\:$the receiver collection efficiency, $\:{\eta\:}_{SMF}\:$the SMF coupling efficiency and $\:{{\upeta\:}}_{turb}$ the loss due to turbulence.

### Atmospheric absorption

To calculate the absorption coefficient for a horizontal link, we used the well-established LOWTRAN software^25^. LOWTRAN can provide atmospheric absorption and scattering over a wide wavelength range, on horizontal or slanted paths, considering both geographical and seasonal atmospheric variations. The absorption coefficient is estimated to be about 0.2 dB/km in the C-band, which corresponds to about 0.1 dB over the 0.5 km FSO link.

### Collection efficiency

The receiver collection efficiency ($\:{\eta\:}_{{R}_{x}}$) accounts for the limited size of the receiver aperture, $\:{D}_{{R}_{x}}$, which collects the incoming beam. In our modelling we also consider how turbulence-induced effects, such as beam broadening, beam wander, and scintillation, influence the collection efficiency η_Rx_.

### Turbulence-induced beam broadening

The beam size $\:w$, as a function of distance z, of a collimated Gaussian beam whose waist equal to w_0_ which propagates in the vacuum is given by:

5 $$\:w\left(z\right)={w}_{0}\sqrt{1+{\left(\frac{\lambda\:z}{\pi\:{w}_{0}^{2}}\right)}^{2}}$$

Turbulence-induced beam broadening refers to the expansion of the optical beam as it propagates through a free space channel with atmospheric turbulence. The turbulence causes fluctuations in the refractive index of the air, which in turn distorts the beam and leads to its spread over a larger area. According to Kolmogorov’s theory the strength of turbulence is parametrized by the refractive-index structure constant $\:{C}_{n}^{2}$, which may be considered constant along a horizontal link. Studies on the turbulence-induced coherence loss on Gaussian beam propagation suggest that it follows the formula:^24^

6 $$\:w\left(z\right)={w}_{0}\sqrt{1+(1+\frac{2{w}_{0}^{2}}{{\rho\:}_{0}^{2}\left(z\right)}){\left(\frac{\lambda\:z}{\pi\:{w}_{0}^{2}}\right)}^{2}},$$

where $\:{\rho\:}_{0}\left(z\right)={\left(1.46{C}_{n}^{2}{k}^{2}z\right)}^{-\:\frac{3}{5}}$ is the plane-wave atmospheric spatial coherence radius and k = 2π/λ is the wavenumber. The average contribution to the collection efficiency caused by diffraction and beam broadening for a receiver with a limited aperture diameter D_Rx_ is given by:

7 $$\left\langle {\eta _{{D_{{R_{x} }} }} } \right\rangle = 1 - e^{{ - \left[ {\frac{{D_{{R_{x} }} ^{2} }}{{2w\left( z \right)^{2} }}} \right]}} ,$$

where it should be noted that pointing errors are omitted for simplicity. The term $\:w\left(z\right)/2$ corresponds to the standard deviation of the Gaussian intensity profile at position z, and D_Rx_ represents the diameter of the circular area. This form ensures that the fraction of energy collected by the receiver is captured as a function of the receiver size and the beam spread.

### Beam wander

The beam size term $\:w\left(z\right)$ that appears in Eq. (6), refers to the average size of the beam at a time interval significantly longer than the characteristic timescales of turbulence dynamics and it is usually referred to as the long-term beam size. This is commonly referred to as the long-term beam size^24^. To account for the instantaneous beam size at a given distance z, one must take into account the variance of beam-wander fluctuations at the receiver aperture plane $\left\langle {r_{c}^{2} } \right\rangle$ which is given by the following equation:

8 $$\left\langle {r_{c}^{2} } \right\rangle = 2.42C_{n}^{2} z^{3} w_{0}^{{ - \frac{1}{3}}}$$

Thus, the beam will have a spot width given by the following relation:

9 $$w_{{short}} \left( z \right) = \sqrt {w\left( z \right)^{2} - \left\langle {r_{c}^{2} } \right\rangle }$$

### Scintillation

Scintillation refers to the rapid fluctuations in the intensity of a light beam as it propagates through a medium with atmospheric turbulence. These variations are caused by the random refractive index changes in the atmosphere, which lead to the beam being focused and defocused at different points along its path. As a result, the beam’s intensity at the receiver can fluctuate significantly. The atmospheric scintillation is measured in terms of the scintillation index σ_Ι_, defined as the normalized variance of the irradiance fluctuations calculated using Eq. (4). Assuming that the random medium is homogeneous, the variations in the refractive index are described by the refractive-index structure parameter $\:{C}_{n}^{2}$. Under weak turbulence conditions ($\:{\sigma\:}_{I}$ << 1) the scintillation index is proportional to the Rytov variance$\:\:{\sigma\:}_{R}$, defined as:

10 $$\:{\sigma\:}_{R}=1.23{C}_{n}^{2}{k}^{\frac{7}{6}}{L}^{\frac{11}{6}},$$

where k is the wavenumber, L is the propagation path length. Finally, the loss due to scintillation $\:{\eta\:}_{scint}$ can be approximated as in^24^:

11 $$\:{\eta\:}_{scint}={\left(1+{\sigma\:}_{I}^{2}\right)}^{-1/4},$$

where $\:{\sigma\:}_{I}$ is defined as previously.

### Single mode fiber coupling

We model the fiber coupling efficiency of a point-to-point FSO link by expressing the coupling efficiency, $\:{\eta\:}_{SMF}$ through Strehl ratio-like metrics, since the latest has been suggested as an estimator for SMF coupling efficiency^26,27^. We also consider the optical coupling efficiency, η_0_, which reflects the mismatch between an unperturbed received optical beam and the Mode Field Diameter (MFD) of the SMF. In detail, the Strehl Ratio (SR) in the presence of atmospheric turbulence is given as follows:

12 $$\:SR={\left[1+\gamma\:{\left(\frac{{D}_{Rx}}{{r}_{0}}\right)}^{5/3}\right]}^{-\frac{6}{5}},$$

where factor γ accounts for the level of Adaptive Optics (AO) correction assuming 1, 0.28, and 0 for no correction, tip tilt correction and full AO correction respectively (no correction in our case), whereas r_0_ is the Fried parameter that characterizes atmospheric turbulence across the horizontal path and is given by Eq. (13):

13 $$\:{r}_{0}=2.1{\left(0.55{C}_{n}^{2}{k}^{2}z\right)}^{\raisebox{1ex}{$-3$}\!\left/\:\!\raisebox{-1ex}{$5$}\right.}$$

Accounting for the efficiency of the receiving telescope, we also model the optical coupling efficiency which is determined by the design optics of the receiving collimator^19^. The ideal coupling efficiency can be parametrized by:

14 $$\:{\eta\:}_{0}={2\left[\frac{\mathrm{exp}\left(-{\beta\:}^{2}\right)-\mathrm{exp}\left(-{\beta\:}^{2}\:{a}^{2}\right)}{\beta\:\:\sqrt{1-{\alpha\:}^{2}}}\right]}^{2},$$

where α, β are given by:

15 $$\:a=\frac{{D}_{obs}}{{D}_{r}},\:{\upbeta\:}\:={\uppi\:}\frac{{\mathrm{D}}_{Rx}}{4\:{\uplambda\:}}\frac{MFD}{f},$$

where D_obs_ is the obscuration aperture diameter which in our case is zero, MFD denotes the mode-field-diameter of an SMF fiber, which is equal to 10.4 μm and $\:f$ the focal length of the receiving collimator. Since the mode characteristic of an SMF can be represented by a Gaussian function and given that the aperture plane and the focal plane are interconnected through a Fourier transform, optimal coupling is possible only when a Gaussian beam matches the aperture within the coupling optics. Nevertheless, the average profile of incoming light tends to exhibit a flat-top distribution which permits a maximum average coupling efficiency of approximately 81% which can be extracted from Eq. (14) for α = 0 and β = 1.12. Finally, η_sys_ accounts for the correction system optical components’ transmittance. The estimated coupling efficiency can then be determined by:

16 $$\:{\eta\:}_{\mathrm{S}\mathrm{M}\mathrm{F}}=SR\:{\eta\:}_{0}{\eta\:}_{\mathrm{s}\mathrm{y}\mathrm{s}}$$

### Noise impairments & filtering techniques

The main noise contributions in our setup arises from the X-talk from the clock and the classical signal, the background sky radiance during the day and Spontaneous Raman Scattering (SpRS) noise due to co-propagation over the same 200 m fiber segment. To mitigate these impairments, spectral and temporal filtering techniques were employed. Band stop filters were used to suppress X-talk while fiber coupling acted as a spatial filter, significantly reducing background sky radiance coupling into SMF, enabling daylight operation. At the receiver, narrow bandpass filters combined with temporal filtering via gated-mode SPAD operation effectively suppressed residual X-talk, Raman, and background noise. X-talk is the dominant source of noise in the system, as classical signals (such as clock and data signals) are co-transmitted alongside quantum signals. The total X-talk intensity reaching the detector, measured in Watts, can be determined using the following formula^28^:

17 $$\:{P}_{xatlk}={P}_{0}\:\eta\:\:{10}^{-\frac{\xi\:}{10}},$$

where $\:{P}_{0}$ is the classical launched intensity (Watt), η corresponds to the total link’s attenuation, and $\:\xi\:$ to the overall suppression ration between the quantum and the classical signal (dB). In our coexistence experiments, several optical intensity values were transmitted from the QTx side, with the maximum being + 10.7 dBm as indicated in Table 1. After approximately 12.5 dB of attenuation introduced by the FSO transmission, this results in about − 1.8 dBm being coupled into the SMF. Given this injected power (representing the worst-case scenario for Raman noise) and the short fiber length (~ 200 m), the contribution of SpRS-generated noise photons remain negligible. More information about the setup used for the SpRS noise measurement can be found in Fig. S5, whereas the SpRS noise profile generated by the 200 m fiber can be also found in Fig. S6 in Supplementary Information Online.

Another significant noise source is background solar radiance, which is particularly high during daylight. The receiver’s background optical power depends on the intensity of this radiance and the optical characteristics of the receiver. In our case, the fiber collimator aperture was relatively small (4.2 cm), reducing the amount of captured noise photons. Due to the fiber’s Field of View (FOV), light arriving from random angles cannot be effectively coupled into it. As a result, the noise that enters the fiber is significantly reduced. Since the receiver focal length $\:f\:$is selected to maximize the optical system efficiency it is considered that^19^:

18 $$\:MFD\:=\:{\beta\:}_{opt}\:\left(\frac{2}{\pi\:}\right)\:\lambda\:\:f/{D}_{Rx}$$

where $\:{\beta\:}_{opt}=1.12$ for our case, since an unobstructed aperture was used. Since the FOV is defined by the incidence angle where the spot on the focal plane is displaced to a distance equal to half of the MFD, we obtain:

19 $$\:FOV\:=\frac{MFD}{2f}=\left(\frac{{\beta\:}_{opt}}{\pi\:}\right)\frac{\lambda\:}{{D}_{Rx}}$$

Therefore, by combining the equations above, one can conclude that the background noise coupled into an SMF is independent of the receiver aperture diameter. Finally, to translate the intensity that is coupled in the fiber into detected noise per second, one needs to include the Bob’s components loss such as filters, polarizations controllers and PBS elements. Therefore, the background noise that reaches the detector can be expressed in count per second as:

20 $$\:{P}_{bac{k}_{cps}}=\frac{{\beta\:}_{opt}^{2}}{4h\:v}\:{\mathrm{B}}_{\lambda\:\:}{{\uplambda\:}}^{3}\varDelta\:\lambda\:{\:\tau\:}_{b}{\tau\:}_{f}\:qe,$$

where $\:hv$ denotes the photon energy, whereas $\:{\tau\:}_{b}$and $\:{\tau\:}_{f}$ correspond to the QRx components transmittance and spectral filter transmittance, respectively while $\:qe$ corresponds to the detector’s quantum efficiency. It is calculated that for a typical background solar radiance during daylight (~ 1.5 W/sr/µm/nm^29^) the noise reaching the detectors after a spectral filtering passband of $\:\varDelta\:\lambda\:=0.1\:nm$ is expected to be negligible (< 10 cps in free running mode). However, some background solar noise was still able to couple into 200 m fiber transmission, since a part of the fiber was directly exposed to sunlight.

### Secure key rate Estimation using decoy-state BB84

In this study, a dual approach is employed by combining theoretical modeling with experimental measurements to evaluate the performance of our QKD system over the 0.5 km FSO link. The methodology involves direct comparison between experimentally measured metrics and those predicted by a software toolbox, which allows us to assess the accuracy of our model in estimating the amount of secure key generation in our implementation. More specifically, the expected SKR is estimated by feeding various experimental parameters from the field trial into the semi-empirical software simulation toolbox. More specifically, the toolbox receives as an input the total channel loss, the background noise level and the system configuration (SPAD characteristics, filters loss, detector visibility, etc.). As an output key metrics for a QKD system, such as the raw click rate, QBER and SKR, are calculated to determine the system’s performance. Below, the theoretical approach for this analysis is provided. For the calculation of the expected SKR the DS-BB84 QKD protocol has been assumed. The lower bound for key generation rate is given by the following formula^30^:

21 $$\:{R}_{sec}={f}_{rep}q\left[-{Q}_{\mu\:}{f}_{ec}\left({{\rm\:E}}_{\mu\:}\right){H}_{2}\left({{\rm\:E}}_{\mu\:}\right)+{Q}_{1}\left(1-{H}_{2}\left({e}_{1}\right)\right)\right]{\eta\:}_{dead},$$

where $\:{f}_{rep}$ is the repetition/emission rate of the source, $\:q$ depends on the implementation (set to 0.9 assuming the efficient BB84), the subscript $\:\mu\:$ denotes the intensity of signal state, $\:{Q}_{\mu\:}$ is the gain of the signal states, $\:{{\rm\:E}}_{\mu\:}$ is the overall QBER, $\:{Q}_{1}$ is the gain of single-photon states, e_1_ is the error rate of single-photon states, $\:{f}_{ec}$ $\:\left(x\right)$ is the bidirectional error correction efficiency and H_2_(x) is the binary Shannon information function given by: $\:{H}_{2}\left(x\right)=-x{log}_{2}\left(x\right)-(1-x){log}_{2}\left(1-x\right)$. The four essential variables required in Eq. (21) are $\:{Q}_{\mu\:}$ and $\:{{\rm\:E}}_{\mu\:}$ and $\:{Q}_{1}$ and e_1_. $\:{Q}_{\mu\:}$ corresponds to the gain of the signal state; and $\:{E}_{\mu\:}$ corresponds to the overall QBER. The values of $\:{Q}_{\mu\:}$ and $\:{E}_{\mu\:}$ can be calculated as:

22 $$\:{Q}_{\mu\:}={Y}_{0}+1-\mathrm{exp}\left(-\eta\:\mu\:\right),$$

23 $$\:{E}_{\mu\:}=\frac{{{e}_{0}{Y}_{0}+\:e}_{det}\:(1-\mathrm{exp}\left(-\eta\:\mu\:\right))}{{Q}_{\mu\:}}$$

where $\:\eta\:={\eta\:}_{channel}{\eta\:}_{QRx}$ corresponds to the overall end-to-end transmittance, $\:{e}_{det}$ corresponds to the baseline system error rate and is equal to (1-V)/2, where V is the detectors’ visibility,$\:\:{e}_{0}$ corresponds to the error rate of the background noise, $\:{e}_{0}$ is set to 1/2 assuming that the background noise is random, and $\:{Y}_{0}$ corresponds to the probability of the detector to click due to dark counts, background noise or after pulsing probability. To determine the signal gain and signal-state QBER the experimentally measured values of η and Υ_0_ are fed into the theoretical model. The validity of the theoretical approach is confirmed since the calculated signal gain and QBER values match the experimentally measured ones. Finally, it is important to note here that Eq. (22) includes the term η_dead_ which accounts for the effects regarding the saturation of the detectors after each detection. Detectors can be kept closed during a short period of time called dead-time, τ_d_, to combat phenomena such as afterpulsing reducing the output rate to a maximum of 1/ τ_d_. Thus, the dead time efficiency is given by:

24 $$\:{\eta\:}_{dead}=\frac{1}{(1+{\tau\:}_{d}{f}_{rep}{P}_{click})},$$

where $\:{P}_{click}$ denotes the probability of the detector to fire due to signal, afterpulsing or noise photons within a gate time window. For the decoy-state protocol assumptions, the mean photon number of the signal and decoy states has been set to 0.65 (in accordance with the experimental value) and 0.1 photons per pulse, respectively. The signal, decoy, and vacuum pulses are assumed to be sent with probabilities of 0.9, 0.05, and 0.05, respectively.

## Conclusion

In conclusion, this research provides experimental evidence of polarization-encoded, C-band QKD over free space channels based on low-cost SMF-coupled optical terminals. A successful BB84-QKD experiment was demonstrated for both daylight/nighttime conditions showing acceptable QBER values which allow the distillation of > 1kbps SKR in the asymptotic regime of DS-BB84 protocol implementation. Leveraging a combination of spatial-/temporal-spectral-domain filtering techniques, we show that our proposed FSO-QKD solution can be also integrated with the deployed fiber infrastructures coexisting with > 10mW classical signals in C-band. With fast and simple installation for FSO terminals in rooftops and robustness against noise-photon contamination arising from the intense data signals propagating over the in-building single-mode fibers, the proposed scheme can provide a practical solution for exchanging quantum keys between end users hosted in different buildings in city-scale networks. Future efforts will focus on further improving system performance, by enhancing resilience against environmental disturbances via stabilization circuitries based on closed-loops and will explore the feasibility of entanglement distribution over the deployed fiber/FSO infrastructure.

## Supplementary Information

Below is the link to the electronic supplementary material.

Supplementary Material 1

## Data Availability

The datasets generated during and/or analyzed during the current study are available from the corresponding author on reasonable request.
