# Supplementary material for: Practical approach to last-mile converged free-space and fiber QKD for secure city-scale networks
Source: Sci Rep. 2026 Jan 28;16:4073. doi: 10.1038/s41598-025-34184-z (PMC12855851; doi:10.1038/s41598-025-34184-z)
Supplement: Supplementary file 1 — Supplementary Material 1 [file 41598_2025_34184_MOESM1_ESM.docx]

Last-mile Free-Space/Fiber QKD: A Practical Approach towards Secure City-scale Networks

Aristeidis Stathis*^,+,1^, Argiris Ntanos**,^+,1^, Panagiotis Kourelias^1^, Evridiki Kyriazi^1^, Panagiotis Toumasis^1^, Nikolaos K. Lyras^2^, Athanasios D. Panagopoulos^1^ , Hercules Avramopoulos^1^, Giannis Giannoulis^+,1^

*^1^National Technical University of Athens, School of Electrical and Computer Engineering, 15780, Athens, Greece*

*^2^Optoelectronics Section, European Space Agency, Estec Keplerlaan 1, 2201 AZ Noordwijk, The Netherlands*

[*stathisaris@mail.ntua.gr](mailto:*ntanosargiris@mail.ntua.gr), [**ntanosarigiris@mail.ntua.gr](mailto:**ntanosarigiris@mail.ntua.gr)
+these authors contributed equally to this work

# Supplementary Methods

The full setup is also depicted in Figure S1 below for completeness.


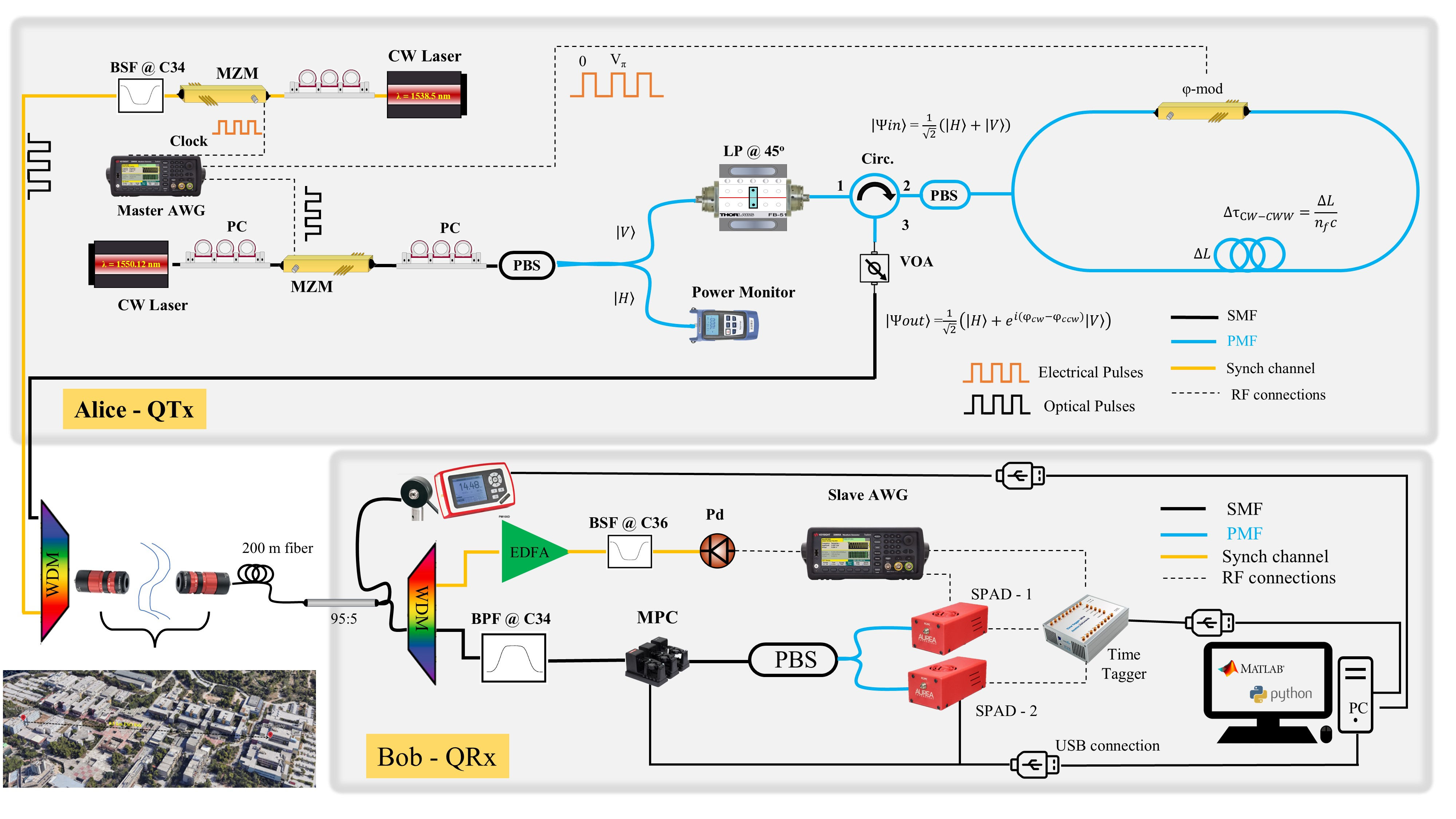


Figure S1. Field trial setup

***QKD in-house polarization encoded source characterization***

The intrinsic QBER (iQBER) of our source along with the Polarization Extinction Ratio (PER) achieved in the receiving setup were measured in a back-to-back configuration and were found to be lower than 1% and more than 25 dB, respectively, for over one hour of continuous operation as indicated by the figures below. The iQBER values shown in Fig. S2 were derived from the Extinction Ratio (ER) of the QTx source for each basis as follows: $iQBER=\frac{1-PER_{QTx}}{1+PER_{QTx}}$.

***Calculation of the scintillation index and Cn^2^***


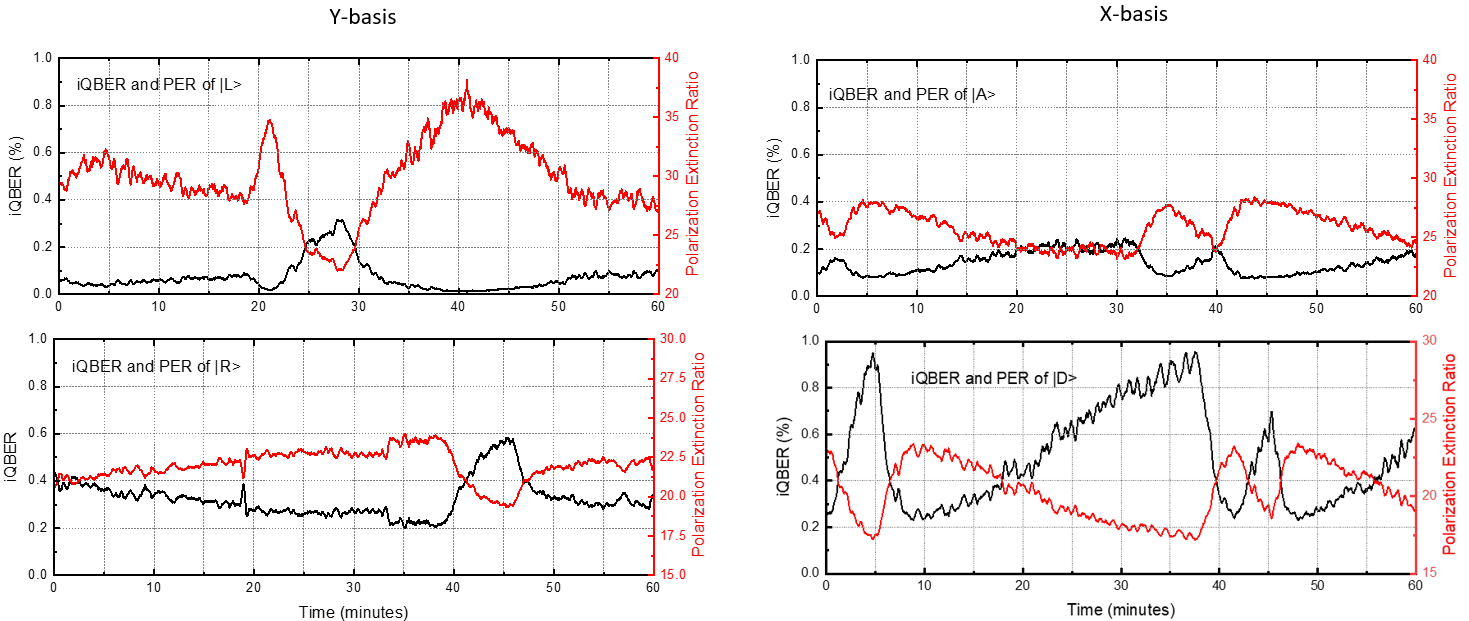


Figure S2. Intrinsic QBER of the custom made QKD polarization encoded source.

In atmospheric turbulence studies for Free-Space Optical (FSO) communication systems, the scintillation index (SI) quantifies the intensity fluctuations of received optical signals. A parameter for the strength of intensity fluctuations is the normalized variance of the intensity—usually called the intensity scintillation index ($\sigma_{I}^{2}$ )[S1] and is given in eq. *(1)*:

$\sigma_{I}^{2}=\frac{{<I}^{2}>}{{<I>}^{2}}-1$ *, (1)*

where (*I*) is the normalized irradiance measured during the operation of the FSO link. The scintillation index can also be evaluated through the Rytov variance ($\sigma_{R}^{2}$), a metric that quantifies the cumulative turbulence effects along the propagation path considering the wavelength (*λ*) of the transmitted signal taking the form of eq. *(2)*:

$\sigma_{R}^{2}=\alpha C_{n}^{2}k^{\frac{7}{6}} L^{\frac{11}{6}}$*, (2)*

where (*a*) depends on the characteristics of the wave, e.g., for plane waves gets the value 1.23, ($C_{n}^{2}$) is the refractive index structure parameter, ($k$) is the optical wavenumber also calculated by $k=\frac{2\pi}{\lambda}$ where ($\lambda$) corresponds to the wavelength of the quantum signal. Moreover, ($L$) is the propagation path length and specifically, in our case, the length of the FSO link in meters (m). After the calculation of the Rytov variance, the conditions under which the FSO link operated can be deduced. A brief categorization is given below.

Weak- turbulence: $\sigma_{R}^{2}< 0.3$

Moderate- turbulence: $0.3 < \sigma_{R}^{2} < 5$ *(3)*

Strong- turbulence: $\sigma_{R}^{2}> 5$

Under weak turbulence conditions an approximation of the S.I. being proportional to the Rytov variance can be followed, i.e., $\sigma_{I}^{2}\sim\sigma_{R}^{2}$. In that case, supposing that the other parameters are known and $\sigma_{I}^{2}=\sigma_{R}^{2}$, the ($C_{n}^{2}$) can be calculated directly by eq. *(4)*.

$C_{n}^{2}=\frac{\sigma_{I}^{2}}{\alpha k^{\frac{7}{6}} L^{\frac{11}{6}}}$ (m^-2/3^), *(4)*

Consequently, the ($\rho_{0}$) can be calculated by eq. *(5)*.

$\rho_{0}=\left( 1.46 C_{n}^{2}k^{2} L \right)^{\frac{-3}{5}}$*, (5)*

The Fried parameter or else the atmospheric coherence width can also be deduced directly from eq. *(7)*.

$\rho_{0}={1.68 \left( C_{n}^{2}k^{2} L \right)}^{\frac{-3}{5}}$, *(6)*

$r_{0}={2.1 \rho}_{0}$*, (7)*

To provide a probabilistic view upon our power intensity measurements of the operating FSO link, two known turbulence models, i.e., the Lognormal and the Gamma-Gamma distributions as proposed by Andrews et al. [S2], were tested upon the normalized irradiance data.

At first, the Generalized Gamma Distribution (GGD) is employed modelling the intensity fluctuations by projecting the shape and scale parameters, i.e., *a*, *b* and *c*, of the regular Gamma Distribution PDF to (*α*) and (*β*) related to the large-scale and small-scale scintillations of the optical wave transmitting through a turbulent medium. The GGD model has been proposed by Andrews et al. [S2] and has been reported for its wide applicability by numerous studies [S3, S4]. The final form of the GGD PDF is shown in eq. (*8*).

The Probability Density Function- PDF of GGD proposed by Andrews et al. [S2] and reported by Ghassemlooy et al. [S5] takes the following form:

$p\left( I \right)= \frac{2{(\alpha\beta)}^{(\alpha+\beta)/2}}{\Gamma(\alpha)\Gamma(\beta)} I^{\frac{\left( \alpha+\beta\right)}{2}-1}K_{\alpha-\beta}\left( 2\sqrt{\alpha\beta I} \right), I>0$*,* *(8)*

where K(x) is the Bessel function and the parameters (*α*) and (*β*) are related to the large-scale and small-scale scintillations and can be derived from the Rytov variance according to eq. (9) and (10).

$\alpha=\left[ exp\left( \frac{0.49\sigma_{R}^{2}}{\left( 1+1.11\sigma_{R}^{12/5} \right)^{7/6}} \right)-1 \right]^{-1}$, *(9)*

$\beta=\left[ exp\left( \frac{0.51\sigma_{R}^{2}}{\left( 1+0.69\sigma_{R}^{12/5} \right)^{5/6}} \right)-1 \right]^{-1}$, *(10)*

Each time the PDF is fitted on the histogram of the experimental data. Afterwards, the scintillation index can also be calculated from eq. (*11*).

$\sigma_{I}^{2}=\frac{1}{\alpha}+\frac{1}{\beta}+\frac{1}{\alpha\beta}$ *,* (*11*)

The second turbulence model used was the Lognormal distribution, following the methodology reported by Giggenbach et al. [S1]. The respective probability density function-PDF is given by eq. (12).

$p\left( I \right)= \frac{1}{I\sigma_{X}\sqrt{2\pi}} exp\left( -\frac{\left( lnI-\mu_{X} \right)^{2}}{2\sigma_{X}^{2}} \right)$*,* (*12*)

, where, its basic parameters, i.e., the variance ($\sigma_{X}^{2}$) and mean ($\mu_{X}$) are expected to remain stable within the fifty second time window and are given in relation to the scintillation index ($\sigma_{I}^{2}$) by the following equations, i.e., eq. (*13*) and (*14*).


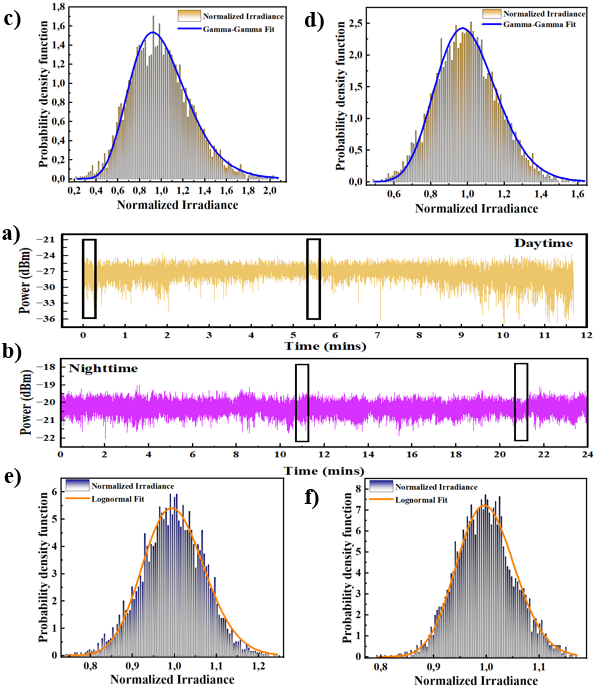


**Figure S3**. a), b) Monitor power over time for the Daytime and Nighttime link respectively, c) ,d) PDF curves of the normalized intensity for daytime conditions and e), f) PDF curves of the normalized intensity for nighttime conditions

$\sigma_{X}^{2}=\ln(1+\sigma_{I}^{2})$*,* (*13*)

$\mu_{X}=-\frac{\sigma_{X}^{2}}{2}$_,_ (*14*)

After the S.I. calculation by taking into consideration the weak turbulence approximation between the S.I. and the Rytov variance, ($\sigma_{I}^{2}\approx\sigma_{R}^{2}$), the ($C_{n}^{2}$), ($\rho_{0}$) and the ($r_{0}$), can be deduced by eq. (*4*), (*5*), (*6*), and (*7*) allowing for a comprehensive analysis of the turbulence's impact on FSO systems. This method combines theoretical approximations with empirical fitting, ensuring robust estimations of turbulence effects under various conditions. During nighttime, a scintillation index ranging between σ_I_^2^=3.1×10^−3^ and σ_I_^2^=5.5×10^−3^ along with the refractive index structure parameter resulting in respective values of Cn^2^=1.36 ×10^−15^ and Cn^2^=2.42×10^−15^ m^-2/3^, indicated weak turbulence and stable signal performance. In contrast, daytime measurements showed a higher scintillation index σ_I_^2^ with values ranging between 0.0284 and 0.0771. Considering the categorization of eq. (3), these values indicate weak turbulence conditions. Accordingly, the values of the refractive index structure parameter (Cn^2^) deduced by eq. (4) spread from 6.16×10^−15^ m^-2/3^ to 1.67×10^−14^ m^-2/3^. The results are plotted and depicted in Fig. S3.

***Wind Speed Calculation at Athens Using ERA5 Data during the field trial***

Hourly wind speed at 10 meters above ground level was computed for the specific geographic location of the field trial (Lat, Lon) based on reanalysis data from the ERA5 dataset, provided by the European Centre for Medium-Range Weather Forecasts (ECMWF) through the Copernicus Climate Data Store (CDS) (S6). ERA5 offers global atmospheric reanalysis at a spatial resolution of ~31 km and an hourly temporal resolution, incorporating observations from satellites, weather stations, aircraft, and buoys using advanced data assimilation techniques (S7). The data used in this study includes the zonal (u) and meridional (v) wind components at 10 m height, specifically for July 4 and 5, 2024, retrieved as hourly fields. These variables represent the wind vector components in the east-west and north-south directions, respectively. To extract the wind speed at the desired location, the dataset was spatially subset to the nearest grid point to the specified latitude and longitude coordinates using bilinear interpolation. Then, the scalar wind speed *U* was calculated using the Euclidean norm of the two components:


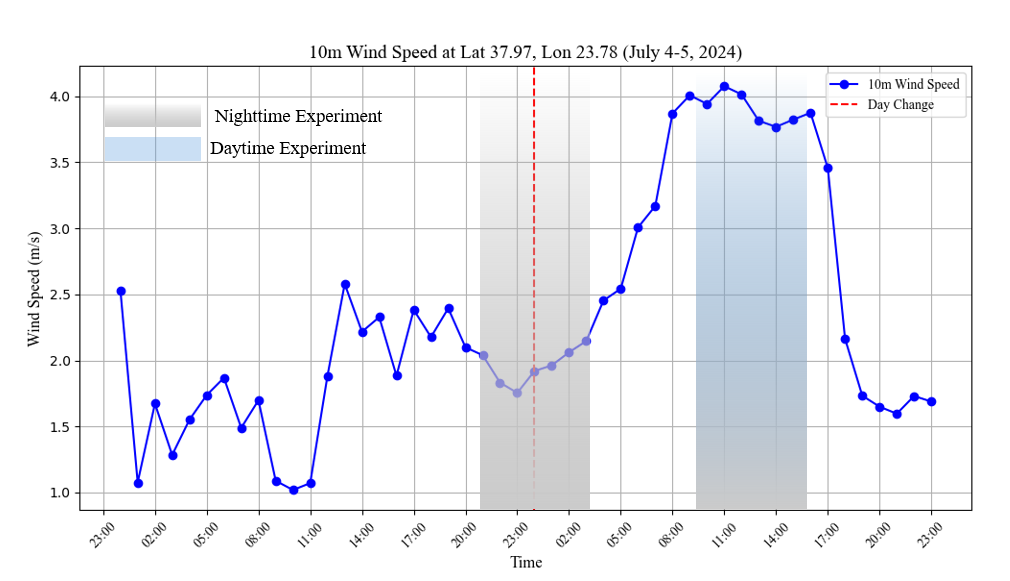


Figure S4. 10m wind speed at the location of the field trial on July 4^th^ and 5^th^ 2024

$U=\sqrt{u^{2}+v^{2}}$​

This method provides an accurate approximation of the instantaneous horizontal wind speed at the 10 m reference height. The resulting time series are visualized in Fig. S4 above to assess the hourly wind speed evolution across the selected dates. The shaded areas correspond to the part of the day where the experimental campaigns took place while the red dotted line corresponds to the date change. As can be seen from the graph the wind speed was almost doubled on 5^th^ of July when compared to that of the previous day. All data processing and visualization were performed using custom made Python scripts, leveraging libraries such as xarray for NetCDF handling, numpy for numerical computation, and matplotlib for plotting.

***Spontaneous Raman noise of the 200m-fiber***

The experimental setup used for the Raman noise characterization in the 200 m fiber segment is depicted in Fig. S5. A triple stage Band-Stop Filter (BSF), comprising of three C34 ITU-grid Dense Wavelength Division Multiplexing (DWDM) 100 GHz modules, with a total Insertion Loss (IL) of 0.8 dB and a Suppression Ratio (SR) of 25 dB was employed before the transmission of the signal over the 200 m fiber segment to minimize the effect of the X-talk noise. To isolate the quantum passband, dedicated at 1550.12 nm, a two-stage Band-Pass Filter (BPF), consisting of a 12.5 GHz spectral slice of a Flexgrid Wavelength Selective Switch (WSS), with IL = 4.5 dB (Finisar DWP-EK-AA, SR = 40 dB) cascaded with a 25 GHz DWDM BPF (OPNETI C34 ITU-grid 25 GHz DWDM, IL = 1.5 dB, SR = 30 dB), was used. To ensure that the Raman noise generated in the 200m fiber was high enough to be clearly detected by the SPADs, we opted for a higher launch optical power level (+5.7 dBm). The Raman noise counts were then extrapolated to the intensity values corresponding to the actual FSO channel attenuation. This approach is valid, since the forward Raman noise has a linear dependence on launched power intensity.

$$P_{ram},f=P_{in}\times e^{-a\cdot L}\times L\times\rho\left( \lambda\right)\times\Delta\lambda$$

Since the X-talk was suppressed close to the DCR levels, it is considered that most of the measured noise counts are generated by the SpRS mechanism. The SPAD was operating at free-running mode and its efficiency and dead time was set to be η = 10% and τ_d_ = 20 μs, respectively, whereas the DCR contribution has been subtracted from the measurements. For the classical channel, a tunable laser source (Yenista Osics TLS Module) was used, propagatingalong the fiber at different wavelengths in the C-band, ranging from 1535 nm to 1565 nm. Figure S6 presents the Raman noise spectral profile for different classical wavelengths allocation assuming that the quantum passband is set to 1550.12 nm. The noise is normalized with respect to the clock signal (set to 1552 nm), which exhibited the lowest Raman noise count rate. Specifically, at a launched intensity of +5dBm at the wavelength allocated for the clock signal the noise rate that was measured at the SPAD was 204 cps, in free running mode (and about 20 cps in gated mode).


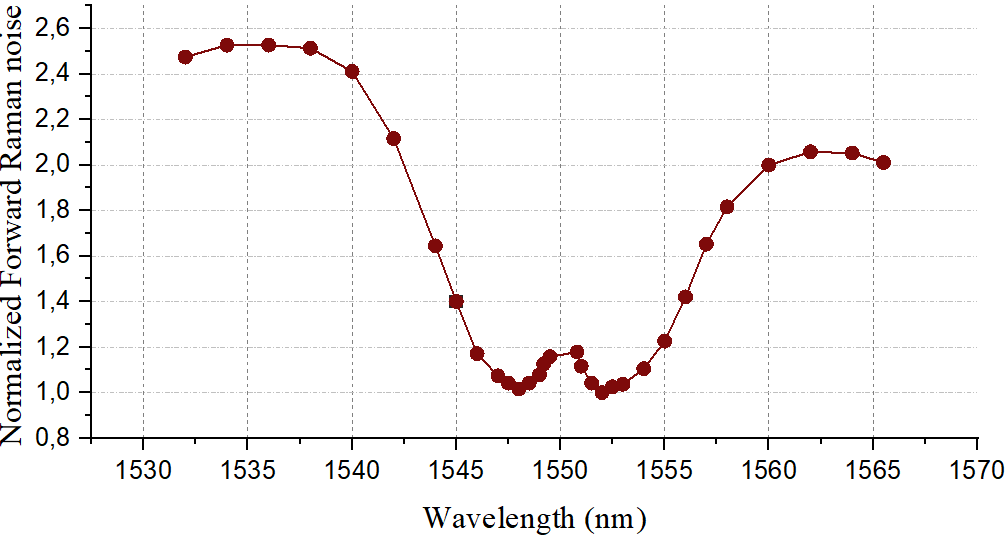


**Figure S6**. Normalized Raman noise profile for different classical channel wavelength allocation, normalized with respect to the clock wavelength (1552nm) where the noise contribution was measured to be minimized.


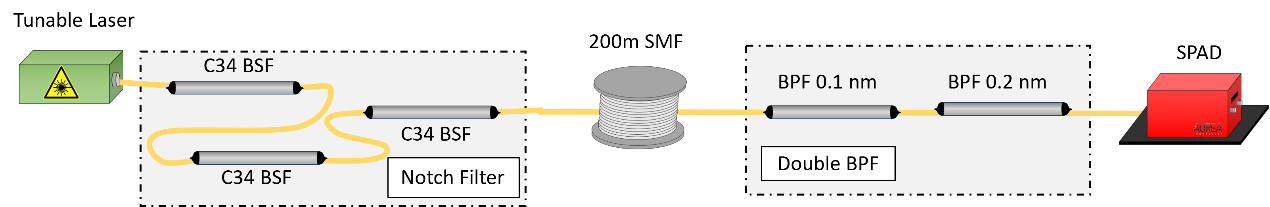


**Figure S5.** Experimental Setup employed for the measurement of the Raman contribution within the 200 m fiber segment.

***References***

S1: Giggenbach, Dirk & Henniger, Hennes. (2008). Fading-loss assessment in atmospheric free-space optical communication links with on-off keying. Optical Engineering. 47. 046001-1. 10.1117/1.2951952.

S2: Andrews, Larry & Philips, R. & Hopen, C.. (2001). Laser Beam Scintillation With Applications. 10.1117/3.412858.

S3: Correia, Vitor & Fernandes, Marco & Monteiro, Paulo & Guiomar, Fernando & Fernandes, Gil. (2024). On the Impact and Mitigation of Turbulence in Fiber-Coupled FSO Systems. IEEE Access. PP. 1-1. 10.1109/ACCESS.2024.3400896.

S4: Rodrigues, Gilberto & Carneiro, Vítor & Cruz, Alberto & Giraldi, Maria. (2013). Evaluation of the strong turbulence impact over free-space optical links. Optics Communications. 305. 42–47. 10.1016/j.optcom.2013.04.058.

S5: S. R. Z. Ghassemlooy and W. Popoola, Optical Wireless Communications: System and Channel Modelling With MATLAB. Boca Raton, FL, USA: CRC Press, 2012, 3.3.6.1 l

S6: Hersbach, H., Bell, B., Berrisford, P., et al. (2020). The ERA5 global reanalysis. Quarterly Journal of the Royal Meteorological Society, 146(730), 1999–2049. https://doi.org/10.1002/qj.3803

S7: Copernicus Climate Change Service (C3S). (2017). ERA5: Fifth generation of ECMWF atmospheric reanalyses of the global climate. <https://cds.climate.copernicus.eu>
